# Severe pediatric asthma therapy: Omalizumab—A systematic review and meta-analysis of efficacy and safety profile

**DOI:** 10.3389/fped.2022.1033511

**Published:** 2023-03-03

**Authors:** Grazia Fenu, Andrea La Tessa, Claudia Calogero, Enrico Lombardi

**Affiliations:** ^1^ Pediatric Pulmonary Unit, “Anna Meyer,” IRCCS Pediatric University-Hospital, Florence, Italy; ^2^ Pediatric Resident, University of Florence, Florence, Italy

**Keywords:** anti-IgE, severe asthma, children, randomized controlled trial, systematic review

## Abstract

**Background:**

Omalizumab is the first biological therapy used to treat moderate-to-severe asthma and certainly the one with the highest number of publications.

**Methods:**

A systematic review and meta-analysis were performed to examine two critical outcomes of omalizumab therapy, asthma exacerbation rate, the reduction of the use of inhaled corticosteroids (ICS), and the improvement of the lung function as a secondary outcome using the following keywords in the MEDLINE database: “anti-IgE, severe asthma, children, and randomized controlled trial.” We specifically selected papers that included moderate-to-severe asthma patients and collected data on children and adolescents.

**Results:**

Four RCT studies (total number of patients = 1,239) were included in the analysis. The reported data on exacerbations showed an overall improvement in the exacerbation rate with a decreased use of inhaled steroids and some other minimal clinically important difference (MCID).

**Conclusions:**

Our systematic review confirms the known findings that omalizumab therapy decreases asthma exacerbation rate and reduces background therapy inhaled steroid dose. Therefore, add-on therapy with omalizumab shows a good efficacy and safety profile, thus proving to be a useful additional therapeutic option.

**Systematic Review Registration:**

https://www.crd.york.ac.uk/prospero/, identifier: CRD42023396785.

## 1. Introduction

Omalizumab, an anti-IgE antibody, has been used to treat adults and adolescents with severe asthma since 2003 and in children aged 6–11 years since 2009.

Asthma is a chronic inflammation that can be differentiated into type 2 (Th2) and non-type 2 (non-Th2) inflammation (1).

Type 2 asthma is characterized by eosinophilic airway inflammation and sensitization, such as IgE-mediated, T helper 2 (Th2)-dependent cytokines, including interleukin (IL)-4, IL-5, and IL-13 (2).

The non-type 2 (non-Th2) asthma is rare in children and adolescents and is characterized by either a neutrophilic or paucigranulocytic pattern promoted by IL-8, IL-17, IL-22, and epithelial cell-derived cytokines (3–6).

Omalizumab is a humanized monoclonal antibody that specifically binds to IgE, preventing it from binding to antigen-presenting cells, mast cells, and basophils. This can help to prevent inflammatory responses or the long-term consequences of allergen exposure, including tissue remodeling, inflammatory cell recruitment, and Th2 inflammation (7).

IgE-type immunoglobulins play a decisive role in the pathogenesis of allergic diseases. After exposure to triggers such as allergens, infectious (especially viral) pollutants trigger a series of IgE-dependent mechanisms.

Therefore, omalizumab by binding to free IgE prevents its binding with its receptor and leads to the formation of inert, nonfunctioning immune complexes (8).

Omalizumab is indicated for treating severe persistent uncontrolled allergic asthma in children aged 6 years and older who are inadequately controlled by high-dose inhaled corticosteroids plus long-acting beta-agonists and who have a positive skin test or in vitro reactivity to a perennial aeroallergen (9).

In adolescents aged >12 years, a reduced forced expiratory volume in 1 s (FEV1) is also required to be less than 80% of the predicted value (9).

From 2009 to 2019, omalizumab was the only biological drug licensed as add-on therapy in children aged ≥6 years with severe allergic asthma not controlled by treatment with high-dose inhaled corticosteroids (ICS) plus long-acting inhaled beta2-agonist (LABA).

The first European Respiratory Society (ERS)/American Thoracic Society (ATS) guidelines on severe asthma in adults and school-age children were published in 2014 (10).

At that time, severe asthma was defined as “asthma that requires treatment with high dose ICS […] plus a second controller (and/or systemic corticosteroids) to prevent it from becoming ‘uncontrolled’ or which remains ‘uncontrolled’ despite this therapy” (10).

Nowadays, there is not a universally accepted definition of severe asthma, and several definitions have been published in different guideline documents (10, 11, 12).

The meeting point in all definitions of severe asthma is poor symptom control despite high-dose ICS treatment (usually budesonide or equivalent ≥400 µg for children younger than 12 years and ≥1,000 µg for older children) (13).

## 2. Methods

**Figure 1:**
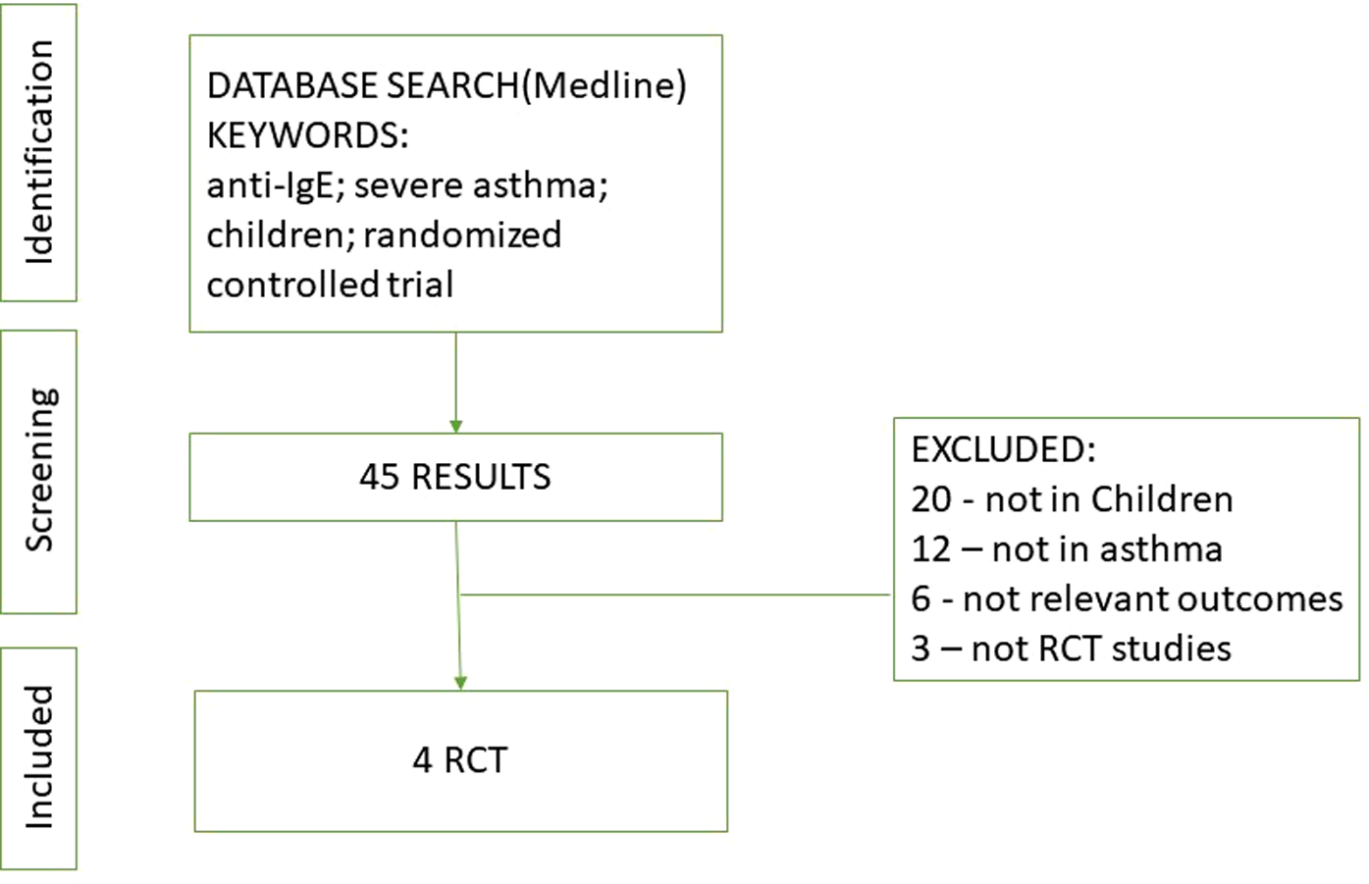
Process identifying studies eligible for the systematic review.

We performed systematic research from the database of MEDLINE among papers written in English using the following keywords: “anti-IgE, severe asthma, children, and randomized controlled trial,” including articles from the earliest records up to October 2022. We included all RCTs conducted in pediatric patients with asthma that compared the efficacy or safety of omalizumab with a placebo or common therapy. This search was further refined using the following inclusion criteria (Figure 1):

1. studies in pediatric patients;
2. studies with a comparison between omalizumab and placebo looking at efficacy and/or safety of omalizumab;
3. studies with the use of omalizumab for asthma; and
4. studies with at least one of the following outcomes: asthma exacerbations, decrease in inhaled corticosteroid dose, and/or drug-related adverse events.

The PRISMA 2020 27-item checklist addressing the introduction, methods, results, and discussion sections was compiled, providing a quality check of the systematic review report (14).

We performed a statistical analysis using Medcalc and Microsoft Excel. We calculated the odds ratio (OR) and 95% confidence interval (CI) for categorical variables. Weighted mean difference and standard deviation (SD) between groups were used for continuous outcomes; the effect on the number of exacerbations was measured by OR analysis and visually represented by a forest plot. The effect on dose-sparing of inhaled steroids was available in two studies and was measured by comparing mean differences; Student *t*-test on unpaired variables was executed to verify the results; results were also represented by a forest plot. Graphs were made with Medcalc.

The safety profile was compiled by summarizing in a table all the adverse events found in three RCTs (15–17) and then categorizing them by systems. Teach et al. (17) used a different categorization by symptoms; to compile the table, we collected them in various systems.

## 3. Results

Four RCTs (including 1,239 pediatric patients) were included (15–18). The characteristics of the included RCTs studies are summarized in Table 1.

**Table 1:** Demographic and clinical characteristics of the selected studies.

Study	Study design	Mean age	Study duration	Asthma severity	Number of patients (omalizumab/placebo group)
Lanier 2009 (15)	Omalizumab add-on/placebo add-on	8,6 (6–12 years)	24 weeks, 52 weeks	Moderate-to-severe	421/206
Busse 2011 (16)	Omalizumab add-on/placebo add-on	8.4 (6–11 years)	24 weeks	Moderate-to-severe	117/120
Teach 2015 (17)	Omalizumab add-on/placebo add-on (third arm with ICS boost)	10,1 (6–12 years)	17–39 weeks	Moderate-to-severe	259/89
Sly 2017 (18)	Omalizumab add-on/placebo add-on	11,5 (6–15 years)	104 weeks	Moderate-to-severe	14/13

### 3.1. Efficacy

**Figure 2:**
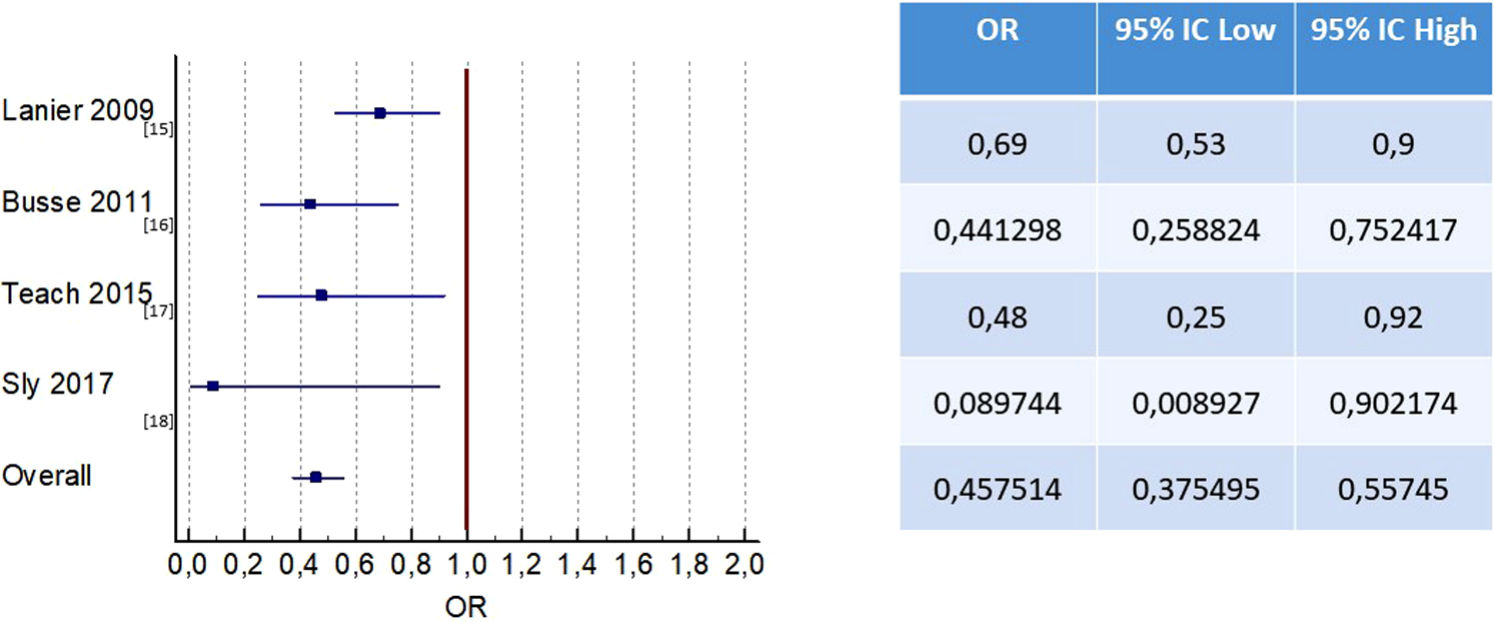
Effect of omalizumab vs. placebo on the number of exacerbations.

The four RCT studies (15–18) that compared omalizumab with. placebo demonstrated a clinical benefit of omalizumab in reducing asthma exacerbations in children with moderate-to-severe persistent asthma (Figure 2).

#### 3.1.1. Exacerbation rate

In the Lanier study that included adolescents with moderate-to-severe allergic asthma, exacerbations were defined as a worsening of symptoms that required doubling of baseline ICS dose and/or rescue treatment with OCS for 3 or more days (15). The risk of exacerbations was reduced by 31% after 24 weeks of treatment with omalizumab when used in conjunction with stable treatment of ICS (15).

When considering the subgroup of patients with severe asthma, as assessed by Kulus et al., the RR was 0.66 (0.44;0.99); this value was considered statistically significant and surpassed the minimal clinically important difference (MCID) of 25% (19).

A study by Busse et al. on 419 children and adolescents who experienced at least one exacerbation found that the rate of exacerbations was 30.3% in the group receiving omalizumab and 48.8% in the placebo group (16).

In a study by Teach et al. on 478 children and adolescents with asthma, those who were treated with omalizumab had a 37% lower risk of exacerbations (defined as a worsening of asthma control requiring oral corticosteroids or hospitalization) over a period of 90 days than those who received a placebo (17).

Finally, in the fourth RTC considered, in the treatment of asthma by omalizumab vs. placebo, it was found that 7% of those in the omalizumab group and 46% of those in the placebo group had experienced a severe exacerbation after 5 months. At the 2-year follow-up, no differences were observed between the two groups. No difference was found in the frequency of moderate exacerbations between the two groups (18).

The rate of asthma exacerbations was an outcome investigated in all four RCTs (15–18).

Of the 620 patients in the placebo group, 54.6% (339 patients) had asthma exacerbations, while in the group receiving omalizumab (1,195 patients), 35.5% of patients had asthma exacerbations. Omalizumab therapy was effective in decreasing the rate of asthma exacerbations compared to placebo [OR 0.44; 95% CI(0.35, 0.56), *P* < 0.001] (Figure 2).

#### 3.1.2. Reduction in ICS use

In the Lanier study, no significant difference was observed in the omalizumab group vs. placebo in the subgroup with severe asthma. The reduction in fluticasone dose from baseline to 52 weeks including both the stable and the steroid adjustment phase was 2.5% in the omalizumab group compared to 2.0% in the placebo group (15).

The Busse study demonstrated a statistically significant difference at the end of the study period, between those receiving omalizumab and placebo, with 663 (SE 23.3) and 771 (23.5) µg budesonide equivalent/day, respectively. This corresponds to a difference of −109 µg/day (95% CI 172; −45), *p* = 0.0012. There was no significant difference between the omalizumab group and the placebo group in terms of moderate dose at the study end (16).

**Figure 3:**
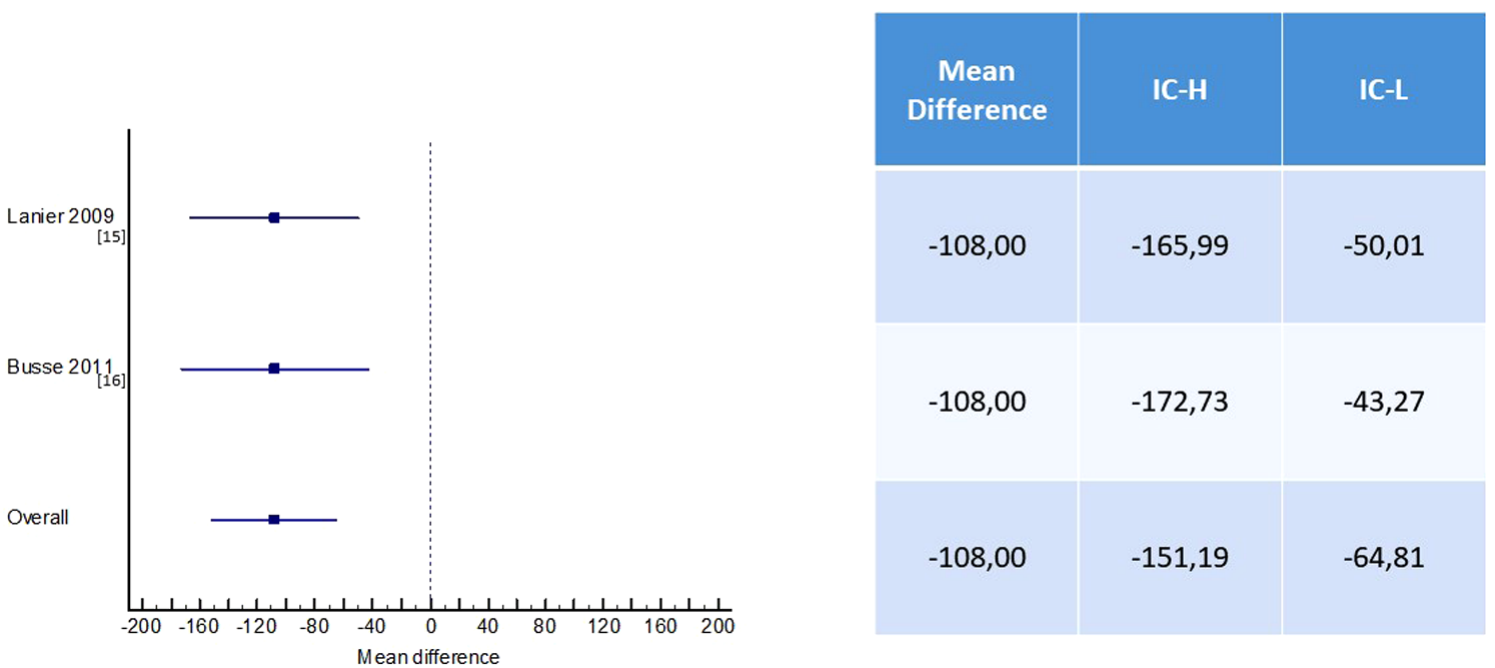
Effect of omalizumab vs. placebo on reduction in ICS use.

The other two studies considered did not designate corticosteroid reduction as an outcome (17, 18); therefore, in our study, we collected data from two studies (15, 16) that reported mean and SD values for the dosage of inhaled corticosteroids. Patients receiving omalizumab had a statistically significant reduction in the required dosage of inhaled corticosteroids compared to the placebo group (mean difference, −108 µg/day, 95% CI −151.19 to −64.81 µg/day, *p* < 0.01) (Figure 3).

### 3.2. Safety

Three (15–17) out of the four studies considered evaluated the safety profile of omalizumab and listed the adverse events of the two groups of omalizumab and placebo; in particular, in Teach et al., the organs involved rather than the individual symptoms are reported (17).

Severe adverse events were counted, and in two (15, 17) of them, no difference was found between omalizumab and placebo groups; in the study by Busse et al., the placebo group had more severe adverse events (30 events) than omalizumab (10 events).

A summary of all adverse events is presented in Table 2.

**Table 2:** Adverse events reported in the included studies.

	Lanier 2009 (15)		Busse 2011 (16)		Teach 2015 (17)		Total	
	Placebo	Omalizumab	Placebo	Omalizumab	Placebo	Omalizumab	Placebo	Omalizumab
Severe adverse events	17	17	30	10	3	3	**50**	**30**
Blood and lymphatic system disorders	–	–	16	1	1	1	**17**	**2**
Eye, ear, and labyrinth disorders	39 (l)	70	7	2	1	6	**47**	**78**
Gastrointestinal disorders	24 (i)	34	2	11	10	19	**36**	**64**
General disorders and administration-site conditions	–	–	8	10	10	63	**18**	**73**
Immune system disorders	–	–	6	1	2	5	**8**	**6**
Infections and infestations	56 (a), 46 (b), 20 (c), 28 (d), 29 (e), 26 (f)	117, 69, 59, 51, 37, 34	26	18	22	63	**253**	**448**
Musculoskeletal and connective tissue disorders	–	–	3	3	1	8	**4**	**11**
Nervous system disorders	33 (g)	58	10	3	9	21	**52**	**82**
Psychiatric disorders	–	–	3	0	2	7	**5**	**7**
Respiratory, thoracic, and mediastinal disorders	25 (h)	44	95	57	11	29	**131**	**130**
Skin and subcutaneous tissue disorders	–	–	24	22	18	41	**42**	**63**
Other	–	–	22	31	4	37	**26**	**68**
Any adverse events	**343**	**590**	**222**	**159**	**97**	**300**	**662**	**1,049**

a, nasopharyngitis; b, URTI; c, pyrexia; d, influenza; e, bronchitis; f, viral URTI; g, headache; h, cough;, i, vomiting; l, sinusitis.

### 3.3. Lung function

From the studies considered in our review, only three studies have looked at the relation between omalizumab and lung function (16–18).

In the study by Busse et al., the difference in predicted FEV_1_% values was 0.92 (95% CI, 0.81;2.64) in the omalizumab group, but it did not reach statistical significance (16).

In the Teach et al. study, the estimated lung function, measured with the predicted FEV_1_% value, was not statistically significant (17).

In an Australian study by Sly et al., lung function was not considered a primary outcome, but the authors concluded that no statistically significant or clinically relevant difference was observed between the two groups when looking at predicted FEV_1_ values (18).

## 4. Discussion

A recent systematic review by the European Academy of Allergy and Clinical Immunology (EAACI) showed that patients with severe asthma reported a reduction to approximately half of the exacerbations and an improvement in other outcomes such as quality of life (QoL) scores and a reduced need for inhaled glucocorticoids to maintain this improved level of asthma control and FEV_1_ (20).

The study by Busse et al. (16) found in 419 participants a reduction in the number of days with asthma symptoms (per 2-week interval: 1.96–1.48 days, with a difference of 24.5%, *P* < 0.001). Another endpoint confirmed a reduction in exacerbations comparing omalizumab with the placebo group (30% vs. 48%); in particular, the percentage of hospitalized patients was 6.3% vs. 1.5%. This study also showed significantly lower doses of inhaled glucocorticoids (*P* < 0.001) and LABA (*P* = 0.003) needed to achieve asthma control.

Omalizumab in patients who were sensitized to cockroach allergen (Bla g1 in house dust ≥2 U per gram) has been demonstrated to be more effective, showing a reduction of 1.1 days with symptoms per 2-week interval (vs. 1.48 of the entire omalizumab group). Also, a greater reduction in the dose of inhaled glucocorticoids and asthma exacerbations was found in those treated with omalizumab compared to the placebo group (16).

Three of the four RCT studies focused on viral infection-induced asthma exacerbations. In both children and adults, asthma exacerbations are indeed often caused by a viral infection (80% of cases) (21–23). Patients with severe asthma are more likely to experience asthma exacerbations caused by respiratory viruses, especially when they have high levels of IgE. The most common virus involved is human rhinovirus (HRV), which is the most commonly detected causative agent in the 5 days prior to exacerbation onset, followed by respiratory syncytial virus (RSV), influenza viruses, parainfluenza viruses, metapneumovirus, bocavirus, adenovirus, and coronavirus (24).

A 2018 Cochrane Review assessing the effects of pharmacotherapy and behavioral interventions to decrease asthma exacerbations in children during the school return in the fall concluded that seasonal omalizumab treatment reduces inflammation but is more effective when combined with other methods (25).

This strategy results in being more expensive with a good safety profile except for injection site pain. This study did not find any data to suggest that this or other seasonal interventions affect asthma control, quality of life, or asthma-related death (25).

A possible explanation for the antiviral role of omalizumab is that it may act by forming IgE/anti-IgE immune complexes. This connection may prevent the interaction of IgE with membrane receptors of plasmacytoid dendritic cells (PD cells) that bind viruses, and this results in the release of interferon-α and activation of the innate immune response (26).

Since asthma has a seasonal pattern of disease activity, with peaks in the spring and fall, a possible change in the efficacy of omalizumab throughout the year has also been studied. The rate of asthma exacerbations doubled during fall and spring in the placebo group while remaining steady in the omalizumab group (4.3% in fall and 4.2% in spring vs. 3.3% in summer).

As for safety, omalizumab is a generally well-tolerated drug. Surveillance on long-term safety reported that the most common adverse events were upper respiratory tract infection and headache (47.1 and 42.7%, respectively), while urticaria occurred in 11 of 225 patients (4.9%) (26).

However, a meta-analysis published in 2021 analyzing more than 1,000 patients included in three RCTs observed the following safety profile: there was no significant difference between placebo and omalizumab groups regarding nasopharyngitis, gastrointestinal disorders, upper respiratory tract infection, skin problem, sinusitis, pyrexia, headache, cough, and influenza (27).

There was also evidence that patients treated with omalizumab experienced less serious adverse events than those who received a placebo. It is worth mentioning that the most serious adverse events were asthma exacerbations requiring hospitalization (27).

## 5. Conclusions

Omalizumab is the first biological therapy that has been used in moderate-to-severe asthma, and it is certainly the one with better evidence of safety and efficacy.

Our systematic review and meta-analysis provide further confirmation that omalizumab reduces the rate of exacerbations and inhaled steroid use in children with moderate-to-severe asthma with a great safety profile.

Its antiviral role is emerging more and more and finds application in pathology such as asthma, where the main actors are viruses, especially in children.

## Data Availability

The original contributions presented in the study are included in the article/**Supplementary Material**, further inquiries can be directed to the corresponding author/s.
