# Supplementary figures and images for: Severe pediatric asthma therapy: Omalizumab—A systematic review and meta-analysis of efficacy and safety profile
Source: Front Pediatr. 2023 Mar 3;10:1033511. doi: 10.3389/fped.2022.1033511 (PMC10020639; doi:10.3389/fped.2022.1033511)

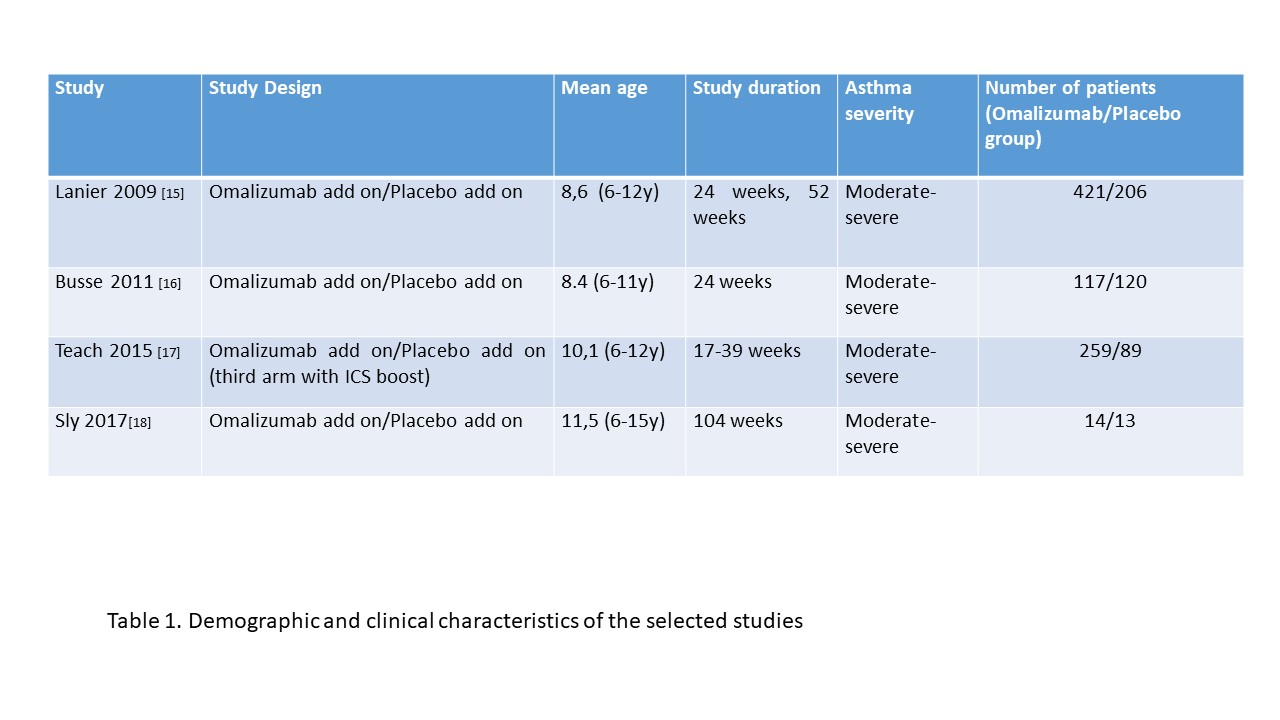

Supplement: Supplementary file 1 [file Image1.jpeg]

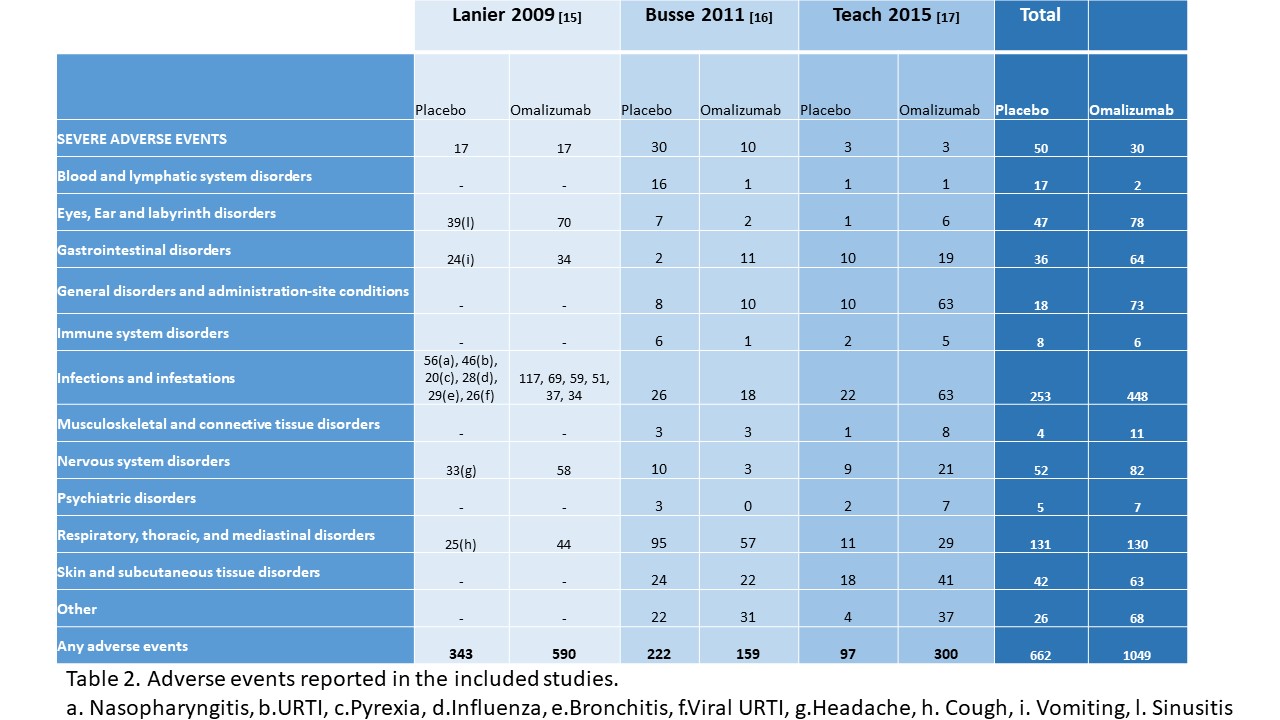

Supplement: Supplementary file 2 [file Image2.jpeg]

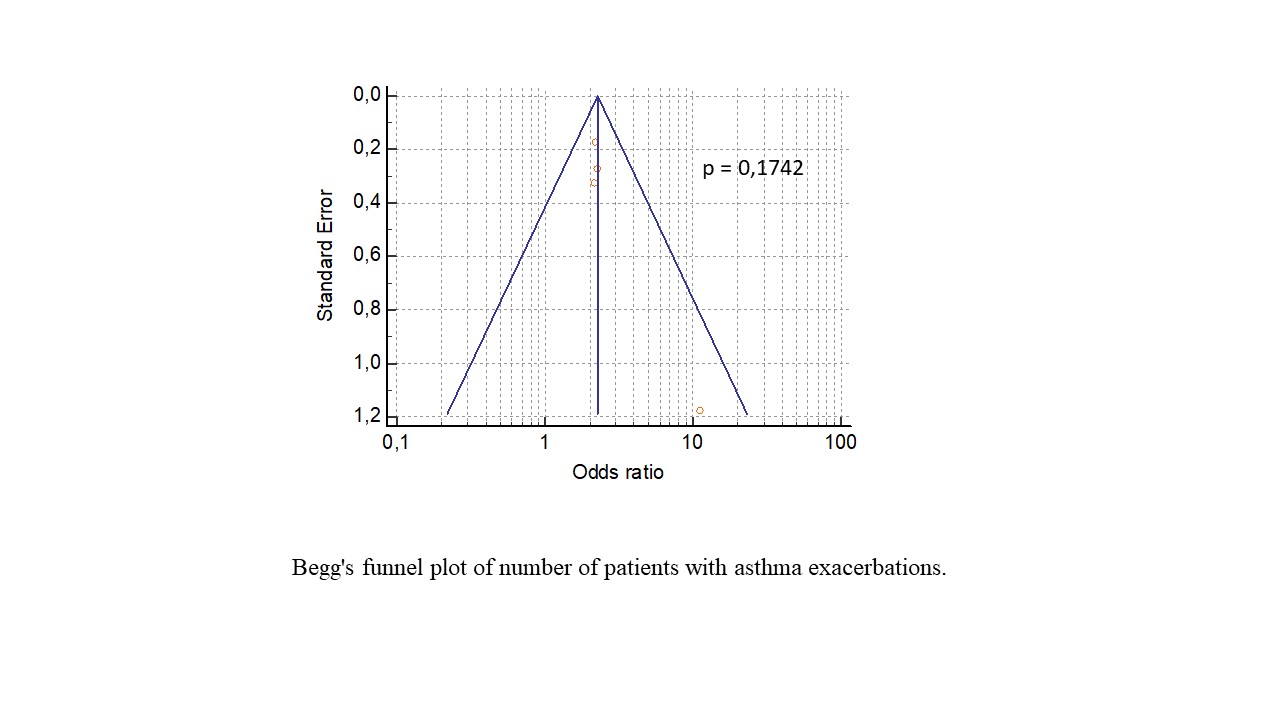

Supplement: Supplementary file 3 [file Image3.jpeg]
